# Publisher’s Announcement

**Published:** 1883-04

**Authors:** 


### PUBLISHER'S ANNOUNCEMENT.

The publisher takes pleasure in announcing to the readers of the REGISTER that he has arranged with Dr. J. H. Logan to take editorial charge of the REGISTER. Dr. Logan is well known to the profession in Georgia and adjacent States, and needs no introduction at our hands.

